# Engineered Wnt7a ligands rescue blood–brain barrier and cognitive deficits in a COVID-19 mouse model

**DOI:** 10.1093/brain/awae031

**Published:** 2024-02-02

**Authors:** Troy N Trevino, Avital B Fogel, Guliz Otkiran, Seshadri B Niladhuri, Mark A Sanborn, Jacob Class, Ali A Almousawi, Benoit Vanhollebeke, Leon M Tai, Jalees Rehman, Justin M Richner, Sarah E Lutz

**Affiliations:** Department of Anatomy and Cell Biology, University of Illinois at Chicago, College of Medicine, Chicago, IL 60612, USA; Department of Anatomy and Cell Biology, University of Illinois at Chicago, College of Medicine, Chicago, IL 60612, USA; Department of Anatomy and Cell Biology, University of Illinois at Chicago, College of Medicine, Chicago, IL 60612, USA; Department of Anatomy and Cell Biology, University of Illinois at Chicago, College of Medicine, Chicago, IL 60612, USA; Department of Biochemistry and Molecular Genetics, University of Illinois at Chicago, College of Medicine, Chicago, IL 60612, USA; Department of Microbiology and Immunology, University of Illinois at Chicago, College of Medicine, Chicago, IL 60612, USA; Department of Anatomy and Cell Biology, University of Illinois at Chicago, College of Medicine, Chicago, IL 60612, USA; Laboratory of Neurovascular Signaling, Department of Molecular Biology, ULB Neuroscience Institute, Université libre de Bruxelles (ULB), Gosselies B-6041, Belgium; Department of Anatomy and Cell Biology, University of Illinois at Chicago, College of Medicine, Chicago, IL 60612, USA; Department of Biochemistry and Molecular Genetics, University of Illinois at Chicago, College of Medicine, Chicago, IL 60612, USA; Department of Microbiology and Immunology, University of Illinois at Chicago, College of Medicine, Chicago, IL 60612, USA; Department of Anatomy and Cell Biology, University of Illinois at Chicago, College of Medicine, Chicago, IL 60612, USA

**Keywords:** blood–brain barrier, SARS-CoV-2, COVID-19, Wnt7a, endothelial cell, neuroinflammation

## Abstract

Respiratory infection with SARS-CoV-2 causes systemic vascular inflammation and cognitive impairment. We sought to identify the underlying mechanisms mediating cerebrovascular dysfunction and inflammation following mild respiratory SARS-CoV-2 infection.

To this end, we performed unbiased transcriptional analysis to identify brain endothelial cell signalling pathways dysregulated by mouse adapted SARS-CoV-2 MA10 in aged immunocompetent C57Bl/6 mice *in vivo*.

This analysis revealed significant suppression of Wnt/β-catenin signalling, a critical regulator of blood–brain barrier (BBB) integrity. We therefore hypothesized that enhancing cerebrovascular Wnt/β-catenin activity would offer protection against BBB permeability, neuroinflammation, and neurological signs in acute infection. Indeed, we found that delivery of cerebrovascular-targeted, engineered Wnt7a ligands protected BBB integrity, reduced T-cell infiltration of the brain, and reduced microglial activation in SARS-CoV-2 infection. Importantly, this strategy also mitigated SARS-CoV-2 induced deficits in the novel object recognition assay for learning and memory and the pole descent task for bradykinesia.

These observations suggest that enhancement of Wnt/β-catenin signalling or its downstream effectors could be potential interventional strategies for restoring cognitive health following viral infections.

## Teaser

Does blood–brain barrier (BBB) dysfunction contribute to cognitive deficits in COVID-19? Trevino *et al.* show that SARS-CoV-2 suppresses Wnt/β-catenin, a critical regulator of BBB integrity. Restoring Wnt/β-catenin improved cognition and protected the brain from leakage of blood proteins and immune cells in a COVID-19 mouse model.

## Introduction

Respiratory viral infections are increasingly recognized to influence neurological function.^1,2^ This has been highlighted in the COVID-19 pandemic, which is associated with neurological deficits. A prominent feature of acute SARS-CoV-2 infection is endothelial inflammation.^3^ Indeed, there is increasing evidence that cerebrovascular endothelial cells can become inflamed and dysfunctional in COVID-19.^3-7^ One major function of brain endothelial cells is to regulate the permeability of the cerebrovasculature to macromolecules and leucocytes, a feature known as the blood–brain barrier (BBB). BBB disruption contributes to neuroinflammation and cognitive impairment. Many groups have reported evidence of BBB disruption in brainstem and hippocampus in COVID-19.^8-16^ However, the extent to which brain endothelial dysfunction contributes to neurological deficits, and the underlying pathways, in acute SARS-CoV-2 have not yet been fully defined. A mouse adapted strain of SARS-CoV-2 (MA10) has been generated by engineering the spike protein to bind to the murine homolog of the viral entry receptor, ACE2, and sequential passage of the mouse-adapted strain through mice.^17^ Respiratory inoculation with SARS-CoV-2 MA10 infects ACE2 expressing cells, yielding acute lung infection.^17^ Recently, MA10 has emerged as a promising tool to study mechanisms of SARS-CoV-2 neuropathogenesis, because it recapitulates features of COVID-19 including cerebral perivascular leucocyte cuffing and neuroinflammation, with greater severity in the aged.^17,18^ We identified neurobehavioural deficits in SARS-CoV-2 mice concordant with BBB disruption and neuroinflammation, including deficits of learning and memory. Using an unbiased transcriptomic approach, we determined that respiratory SARS-CoV-2 infection dysregulated brain endothelial Wnt/β-catenin signalling, a critical regulator of BBB function. Targeted delivery of an engineered Wnt7a ligand to the brain vasculature prevented BBB disruption and restored neurobehavioural function in SARS-CoV-2 infection.

## Materials and methods

The mouse adapted SARS-CoV-2 MA10 strain^17^ was propagated and titred on Vero-E6 cells expressing ACE2 and TMPRSS2 (ATCC, CRL1586). Twelve-month-old male C57Bl/6 mice (Jackson Laboratories) were studied. AAV-PHP.eB-Wnt7a^K190A^-P2A-EGFP (AAV-PHP.eB-Wnt7a^K190A^) and AAV-PHP.eB-EGFP^19^ (AAV:Vector) were administered by retro-orbital injection of 2 × 10^11^ viral genomes in 25 µl of PBS 18 days prior to inoculation with SARS-CoV-2 MA10. SARS-CoV-2 MA10 was delivered by intranasal inoculation with 1 × 10^4^ foci-forming units (FFU) MA10 or vehicle in animal BioSafety Level 3 facilities. Behavioural testing (novel object recognition, pole descent), euthanasia, and cell and tissue harvesting was at 5 days post-inoculation with SARS-CoV-2. Animal studies were approved by the Animal Care Committee (20–107; 20–160). Brain microvessels were isolated using gradient centrifugation and dissociated to single cells. Endothelial cells were isolated with positive and negative selection. Endothelial RNA was used for bulk RNA-sequencing. Differential expression analysis was performed between testing groups using DESeq2. ClusterProfiler was used for over-representation analysis of the differentially expressed genes against the gene ontology database. Histology was conducted on formalin-fixed paraffin-embedded sections.

Expanded methods are provided in the Supplementary material online.

## Results

### SARS-CoV-2 induces BBB permeability and cognitive dysfunction

SARS-CoV-2 infection has been shown to increase BBB permeability and neuroinflammation in patients and in animal models. However, the extent to which BBB contributes to neurobehavioural signs of disease is unknown. Therefore, we evaluated the impact of SARS-CoV-2 infection on BBB permeability and behaviour in 12-month-old mice 5 days post infection with SARS-CoV-2 (MA10)^17^ to mimic the more severe presentation associated with age.^4,20^

We initially evaluated clinical features of disease. As previously reported, MA10 inoculation caused weight loss and high levels of viral RNA in the lungs (Supplementary Fig. 1A and B).^17,18^ We detected viral RNA in the brain in a subset of animals, although these values were near the limit of detection: ∼50 copies RNA per mg tissue (Supplementary Fig. 1B). However, no infectious virus was recovered from brains of MA10 infected mice (Supplementary Fig. 1C). These data suggest that active viral replication in the CNS is not a feature of SARS-CoV-2 MA10 infection.

**Figure 1:**
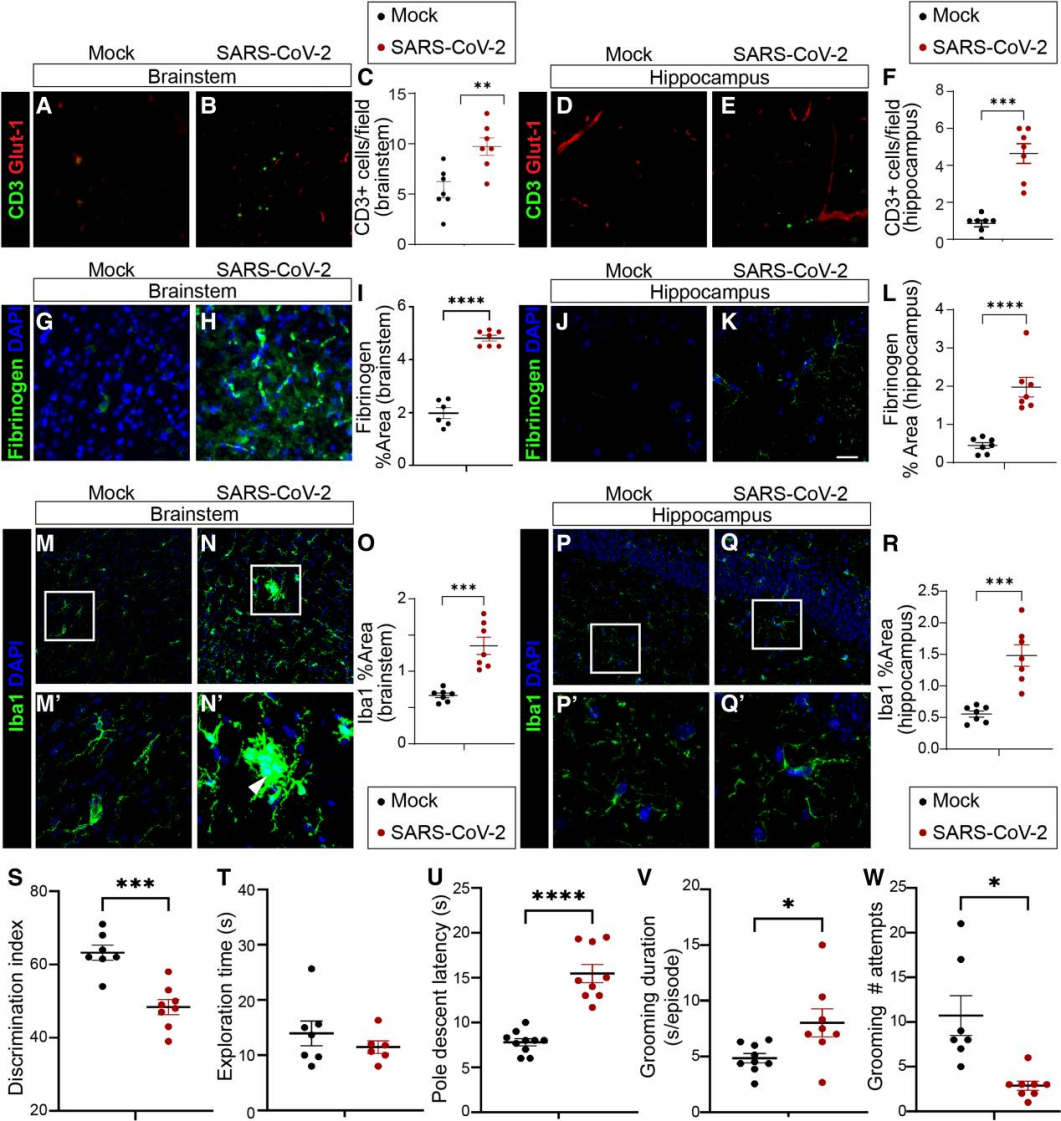
**Blood-brain barrier permeability, neuroinflammation and neurobehavioural changes in SARS-CoV-2 infected mice.** (**A**–**F**) Immunofluorescent staining for CD3+ T cells (green) in brainstem (**A**–**C**) and hippocampus (**D**–**F**) of mock or SARS-CoV-2 infected mice at 5 days post inoculation. Glut-1 (red) used to visualize endothelial cells. CD3+ T cells are more abundant in SARS-CoV-2 infected mice in (**C**) brainstem (*P* < 0.01) and (**F**) hippocampus (*P* < 0.001), unpaired *t*-test. Each dot represents the average value obtained from two to three tissue sections per mouse. Line indicates group mean. Monochromatic images are provided in Supplementary Fig. 6. (**G**–**L**) Immunofluorescent staining for fibrinogen (green) and DAPI (blue) in brainstem (**G**–**I**) and hippocampus (**J**–**L**) of mock or SARS-CoV-2 infected mice. Fibrinogen was more abundant in (**I**) brainstem (*P* < 0.0001) and (**L**) hippocampus (*P* < 0.0001) of SARS-CoV-2 infected mice, unpaired *t*-test. (**M**–**R**) Immunofluorescent staining for Iba1 (green) and DAPI (blue) in brainstem (**M**–**O**) and hippocampus (**P**–**R**) of mock or SARS-CoV-2 infected mice. The white box indicates the region of magnification. Arrowhead (**N'**) indicates a microglial nodule. Significantly greater Iba1+ area in (**O**) brainstem (*P* < 0.001) and (**R**) hippocampus (*P* < 0.001) in SARS-CoV-2 infected mice, unpaired *t*-test. (**S**) SARS-CoV-2 infected mice have significantly lower novel object discrimination in the novel object recognition task for learning and memory than do mock-infected mice (*P* < 0.001, unpaired *t*-test). Discrimination index of 50% indicates novel object preference equal to chance. (**T**) Total object exploration time in the novel object recognition task does not differ between SARS-CoV-2 and mock infected mice, supporting that poor discrimination index in SARS-CoV-2 infected mice is not driven by lack of interest in objects or motility to reach objects. (**U**) SARS-CoV-2 infected mice are significantly slower than mock infected mice to complete the pole descent task for complex motor coordination (*P* < 0.0001, unpaired *t*-test). (**V**) Bouts of spontaneous grooming have longer duration in SARS-CoV-2 infected mice (*P* < 0.05, unpaired *t*-test). (**W**) Fewer individual bouts of spontaneous grooming in SARS-CoV-2 infected mice (*P* < 0.05, unpaired *t*-test).

Cognitive, affective, and autonomic psychomotor impairments are frequent in middle-aged adults with COVID-19.^4,20^ We therefore interrogated the BBB in regions affected in COVID-19 including hippocampus and brainstem. Two markers of BBB dysfunction are leucocyte infiltration and high levels of blood proteins in the brain. We found that SARS-CoV-2 infection increased parenchymal CD3+ T cell density (Fig. 1A–F) and fibrinogen extravasation (Fig. 1G–L) in the hippocampus and brainstem. T cells and fibrinogen within the CNS can promote proinflammatory and neurotoxic microglial activation.^21,22^ We therefore investigated microglial area. We found that SARS-CoV-2 infection increased Iba1+ area in hippocampus and brainstem (Fig. 1M–R), consistent with reports of microglial activation in COVID-19 models.^15,18,23-26^

A complication of acute SARS-CoV-2 infection in humans is neurological impairment.^2-4,7,20^ However, the extent that respiratory SARS-CoV-2 MA10 infection causes neurobehavioural deficits in mice is unknown. To evaluate learning and memory, we measured novel object recognition (Fig. 1S), a task that requires hippocampus. We chose novel object recognition because this assay was compatible with biosafety limitations on work with SARS-CoV-2 infected mice. Importantly, we found that infection disrupted learning and memory (Fig. 1S). We validated that the object exploration time was similar in the familiar phase and in the novel phase in mock infected and in SARS-CoV-2 infected mice (Fig. 1T). These data support the interpretation that changes in novel object recognition were not due to locomotion impairment or inactivity. We then focused on the pole descent assay, in which the mouse turns upside down and descends a pole to return to the home cage. The brainstem contributes to pole descent performance. We found that SARS-CoV-2 infected mice descended more slowly than mock-infected mice (Fig. 1U). We also noted changes in spontaneous self-grooming suggestive of affective changes in infected mice (Fig. 1V and W). Overall, our novel data demonstrate that SARS-CoV-2 MA10 induces neurobehavioural deficits that correlate with BBB permeability.

### SARS-CoV-2 dysregulates the Wnt/β-catenin pathway in brain endothelial cells

**Figure 2:**
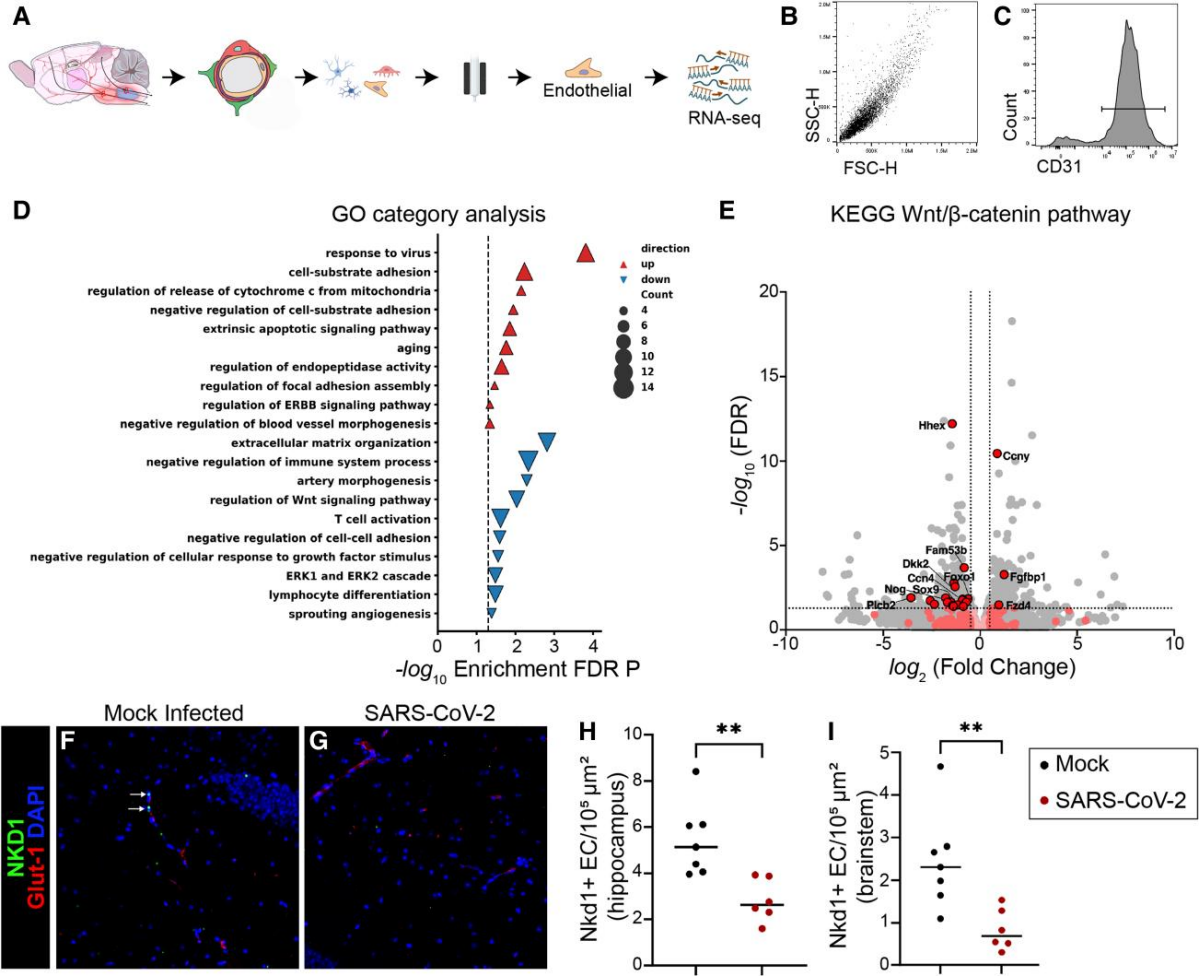
**Dysregulation of brain endothelial cell Wnt/β-catenin signalling in SARS-CoV-2 infection.** (**A**) Schematic depiction of brain microvascular endothelial cell isolation protocol using gradient centrifugation and positive/negative selection 5 days after respiratory inoculation with SARS-CoV-2. (**B**) Flow cytome­­tric analysis of isolated brain endothelial cells demonstrating uniform population for side-scatter (SSC-H) and forward scatter (FSC-H). (**C**) Flow cytometric analysis showing histogram for CD31 out of all cells in **B**. Ninety-four per cent of cells were positive for the endothelial cell marker CD31. (**D**) Gene ontology (GO) category analysis of differentially expressed genes in brain endothelial cells of mice with SARS-CoV-2 as compared to mock infection. (**E**) Volcano plot in which differentially expressed genes in KEGG Wnt/β-catenin pathway are depicted in red. Non-differentially expressed KEGG Wnt/β-catenin genes are depicted in pink. Grey dots represent transcripts that are not part of the KEGG Wnt/β-catenin pathway. (**F** and **G**) Immunostaining for the β-catenin transcriptional target Nkd1 (green) in brain sections of SARS-CoV-2 or mock-infected mice. Brain endothelial cells are visualized with Glut-1 (red). Nuclei are visualized with DAPI (blue). Monochromatic images are provided as Supplementary Fig. 3. (**H** and **I**) Quantification of density of Nkd1+ endothelial cells (EC) in the (**H**) hippocampus and (**I**) brainstem of SARS-CoV-2 or mock-infected mice. Each dot represents the average value obtained from two to three tissue sections per mouse. Line indicates group mean. Unpaired Student’s *t*-test, **P < 0.01. FDR = false discovery rate; KEGG = Kyoto Encyclopedia of Genes and Genomes.

Brain endothelial cells are the central effector cells of the BBB, and control permeability of the CNS to potentially proinflammatory macromolecules and cells from the blood. Therefore, we conducted unbiased analysis of brain endothelial cells to identify pathways that could contribute to the BBB dysfunction and neurobehavioural changes observed in SARS-CoV-2 infection. We conducted RNA-seq on brain endothelial cells of mice with and without SARS CoV-2 infection at 5 days post infection (Fig. 2A–C). For this, we isolated brain microvessels by gradient centrifugation and conducted positive and negative selection, yielding a debris-free population with ~95% immunoreactivity for CD31 (Fig. 2A–C).

SARS-CoV-2 infection altered the brain endothelial cell transcriptome. SARS-CoV-2 infection upregulated gene expression pathways related to response to virus, cell substrate adhesion, and apoptosis, and downregulated pathways related to extracellular matrix organization and immunosuppression (Fig. 1D and Supplementary Fig. 2). Importantly, we found that SARS-CoV-2 infection regulated genes in the Wnt signalling pathway in brain endothelial cells (Fig. 1D). This finding was important because the Wnt/β-catenin signalling pathway is required for BBB integrity.^19,27-30^ Indeed, we have previously shown that inhibition of the Wnt/β-catenin pathway in CNS endothelium exacerbates BBB permeability and worsens clinical presentation of the multiple sclerosis model experimental autoimmune encephalitis.^29^ Canonical Wnt ligands activate a signalling cascade resulting in β-catenin dependent transcription of target genes.^19,27^ Further analysis in the Kyoto Encyclopedia of Genes and Genomes (KEGG) Wnt/β-catenin signalling pathway (Fig. 1E) highlighted changes in SARS-CoV-2 infection including Ccn4/WISP1, transcriptional coactivators and effectors Sox9 and Foxo1, and pathway regulator Dkk2 (Fig. 1E). Of all targets in the KEGG Wnt/β-catenin signalling pathway, 17 genes were significantly downregulated and three were significantly upregulated by SARS-CoV-2 (Fig. 1E). As a validation of changes in the Wnt/β-catenin signalling pathway in brain endothelial cells, we assessed a downstream transcriptional target of canonical β-catenin transcription, Nkd1, at the protein level and found concordant downregulation in hippocampus and brainstem (Fig. 1F–I). Together, these data suggest that the Wnt/β-catenin signalling pathway is dysregulated in brain endothelial cells following SARS-CoV-2 infection.

### Cerebrovascular-targeted Wnt ligands prevent BBB leakage and microglial activation caused by SARS-CoV-2

As we found that SARS-CoV-2 infection dysregulated the Wnt/β-catenin pathway in correlation with BBB dysfunction, we next explored the extent to which enhancing brain endothelial Wnt/β-catenin pathway activity could improve BBB function, neuroinflammation and neurological signs of SARS-CoV-2 infection. To do this we chose an agonist-based approach. Our rationale was that because Wnt7a receptors Gpr124, Reck, Fzd4, and Lrp4/5^19,27^ were not among the genes downregulated by SARS-CoV-2 infection (Fig. 2E), brain endothelial cells in infected mice might retain responsiveness to receptor agonism. We deployed the cerebrovascular-tropic gene therapy vector AAV:PHP.eB to deliver Wnt7a engineered with a lysine to alanine mutation at position 190 (Wnt7a^K190A^) to specifically activate Gpr124/Reck.^19^ Gpr124/Reck is the brain endothelial Wnt7a co-receptor; therefore Wnt7a^K190A^ has increased specificity for brain endothelial cells. We previously demonstrated that AAV-PHP.eB-Wnt7a^K190A^ did not impact behaviour or BBB in healthy adult mice, and prevented BBB permeability in mouse models of glioblastoma and ischaemic stroke.^19^ We therefore administered to mice AAV-PHP.eB-Wnt7a^K190A^-P2A-EGFP (AAV-PHP.eB-Wnt7a^K190A^) or AAV:PHP.eB-EGFP (AAV:vector) prior to inoculation with SARS-CoV-2. Wnt/β-catenin pathway signalling in brain endothelial cells was upregulated ∼3-fold by the engineered ligands (Supplementary Fig. 3), increasing brain endothelial Wnt/β-catenin activity to levels slightly higher than age-matched mice without SARS-CoV-2 (Fig. 2F–I). Weight loss was similar between groups during the first 4 days of infection (Supplementary Fig. 4). A significant improvement in weight emerged at 5 days post infection in mice treated with AAV-PHP.eB-Wnt7a^K190A^, indicating better body condition in response to treatment (Supplementary Fig. 4).

**Figure 3:**
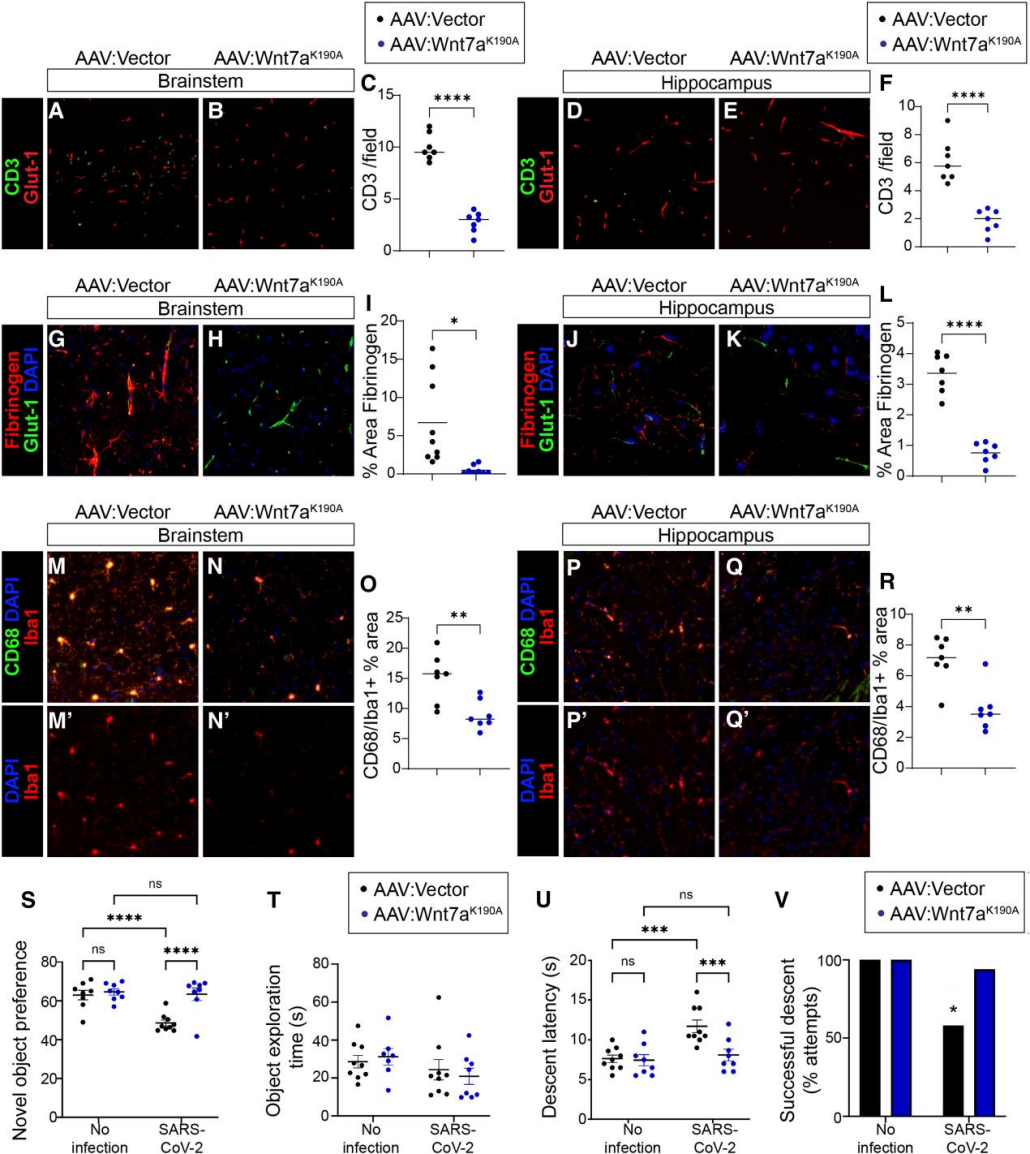
**Cerebrovascular-targeted engineered Wnt7a^K190A^ protects against neurobehavioural impairment, neuroinflammation and blood–brain barrier leakage in SARS-CoV-2 infection**. Mice were treated with AAV-PHP.eB vector control or AAV-PHP.eB-Wnt7a^K190A^ 18 days prior to inoculation with SARS-CoV-2. Behavioural assessment and tissue collection was conducted 5 days after SARS-CoV-2 inoculation. (**A**–**F**) Immunostaining for CD3+ T cells (green) in brainstem (**A**–**C**) and hippocampus (**D**–**F**) of SARS-CoV-2 infected mice treated with AAV-PHP.eB-Wnt7a^K190A^ or AAV-PHP.eB vector control. Glut-1 (red) was used to visualize endothelial cells. SARS-CoV-2 infected AAV-PHP.eB-Wnt7a^K190A^ treated mice had significantly lower density of CD3+ T cells than did AAV-PHP.eB vector control treated mice in brainstem (**C**) and in hippocampus (**F**). Each dot represents the average value obtained from two to three tissue sections per mouse. Line indicates group mean. Unpaired Student’s *t*-test, ****P < 0.0001. (**G**–**L**) Immunostaining for fibrinogen (red) in brainstem (G–I) and hippocampus (**J**–**L**) of SARS-CoV-2 infected mice treated with AAV-PHP.eB-Wnt7a^K190A^ or AAV-PHP.eB vector control. Glut1 (green) was used to visualize endothelial cells. SARS-CoV-2 infected AAV-PHP.eB-Wnt7a^K190A^ treated mice had significantly lower perivascular fibrinogen than did AAV-PHP.eB vector control treated mice in brainstem (**I**; unpaired *t*-test with Welch’s correction for unequal variance, *P < 0.05) and in hippocampus (**L**, unpaired *t*-test, ****P < 0.0001). Each dot represents the average value obtained from two to three tissue sections per mouse. Line indicates group mean. (**M**–**R**) Immunostaining for CD68 (green), Iba1 (red), and DAPI (blue) in brainstem (**M** and **N**) and hippocampus (**P** and **Q**) of SARS-CoV-2 infected mice treated with AAV-PHP.eB-Wnt7a^K190A^ or AAV-PHP.eB vector control. (**O** and **R**) Quantification of percent Iba1^+^ area that is positive for CD68. SARS-CoV-2 infected AAV-PHP.eB-Wnt7a^K190A^ treated mice had significantly lower CD68/Iba1 than did AAV-PHP.eB vector control treated mice in brainstem (**O**; unpaired *t*-test, **P < 0.01) and in hippocampus (**R**; unpaired *t*-test, **P < 0.01). Each dot represents the average value obtained from two to three tissue sections per mouse. Line indicates group mean. Monochromatic images are provided in Supplementary Fig. 6. (**S**) The novel object recognition test for learning and memory was conducted in mice treated with AAV-PHP.eB-Wnt7a^K190A^ or AAV-PHP.eB vector control without or with SARS-CoV-2 infection. There was a Treatment [*F*(1,29) = 13.05, *P* = 0.0011], SARS-CoV-2 [*F*(1,29) = 11.75, *P* = 0.0018], and Treatment × SARS-CoV-2 interaction [*F*(1,29) = 8.397, *P* = 0.0071] for novel object preference. *Post hoc* analysis revealed that SARS-CoV-2 infection impaired novel object recognition in mice treated with the vector control (****P < 0.0001) but not in mice treated with AAV-PHP.eB-Wnt7a^K190A^ (*P* = 0.7145). Indeed, SARS-CoV-2 infected mice treated with AAV-PHP.eB-Wnt7a^K190A^ performed significantly better than SARS-CoV-2 infected mice treated with the control vector (****P < 0.0001). (**T**) Total object exploration was not significantly influenced by Treatment [*F*(1,29) = 0.009622, *P* = 0.9225], SARS-CoV-2 [*F*(1,29) = 2.679, *P* = 0.1125], or Treatment × SARS-CoV-2 interaction [*F*(1,29) = 0.4577, *P* = 0.5041], supporting that the novel object recognition task was not confounded by lack of interest or impaired motility. (**U**) Pole descent latency, a measure of complex motor coordination impairment, was significantly influenced by Treatment [*F*(1,30) = 7.855, *P* = 0.0088], SARS-CoV-2 [*F*(1,30) = 12.10, *P* = 0.0016], and Treatment × SARS-CoV-2 interaction [*F*(1,30) = 6.410, *P* = 0.0168]. *Post hoc* analysis revealed the difference was driven by impairment in the vector-treated SARS-CoV-2 group as compared to the AAV-PHP.eB-Wnt7a^K190A^ treated group (***P < 0.001) or the uninfected group (***P < 0.001). (**V**) SARS-CoV-2 infected mice treated with AAV-PHP.eB-Wnt7a^K190A^ fell off the pole significantly fewer times than those treated with AAV-PHP.eB vector control (Fisher’s exact test, *P = 0.0198).

We first evaluated whether AAV-PHP.eB-Wnt7a^K190A^ prevented BBB dysfunction in SARS-CoV-2 infection. We therefore measured lymphocyte and fibrinogen extravasation in hippocampus and brainstem. We observed ∼80% fewer CD3+ T cells in the brain parenchyma in hippocampus and brainstem in mice that received AAV-PHP.eB-Wnt7a^K190A^ as compared to those receiving vector control prior to SARS-CoV-2 (Fig. 3A–F and Supplementary Fig. 6E–H). Furthermore, we observed a significant reduction in CD8+ T cells in hippocampus and brainstem in mice that received AAV-PHP.eB-Wnt7a^K190A^ as compared to those receiving vector control prior to SARS-CoV-2 (Supplementary Fig. 5). AAV-PHP.eB-Wnt7a^K190A^ also reduced perivascular fibrinogen in the hippocampus and brainstem as compared to mice receiving vector control prior to SARS-CoV-2 (Fig. 3G–L and Supplementary Fig. 6I–L). One consequence of disrupted BBB barrier is microglial activation, which can be assessed by CD68 expression. Indeed, CD68+ microglial area was reduced in hippocampus and brainstem in mice that received AAV-PHP.eB-Wnt7a^K190A^ as compared to those receiving vector control prior to SARS-CoV-2 (Fig. 3M–R and Supplementary Fig. 6M–P). These data indicate that cerebrovascular-targeted Wnt7a prevents BBB leakage and microglial activation caused by SARS-CoV-2.

### Cerebrovascular-targeted engineered Wnt7a^K190A^ significantly improved neurobehaviour after SARS-CoV-2 infection

We next evaluated whether cerebrovascular-targeted Wnt7a^K190A^ might offer protection against neurological signs induced by SARS-CoV-2 in mice. Importantly, AAV-PHP.eB-Wnt7a^K190A^ mitigated the learning and memory deficit caused by SARS-CoV-2 infection, as assessed by the novel object recognition task (Fig. 3S). We validated that the object exploration time was similar in AAV-PHP.eB-Wnt7a^K190A^ or vector control treated SARS-CoV-2 infected mice (Fig. 3T). These data support the interpretation that changes in novel object recognition were not due to locomotion impairment or inactivity. We then focused on the pole descent assay. We found that AAV-PHP.eB-Wnt7a^K190A^ mitigated SARS-CoV-2 induced deficits in the pole descent task (Fig. 3U and V). Overall, our novel data demonstrate that SARS-CoV-2 MA10 induces neurobehavioural deficits that correlate with BBB permeability, and that repairing the BBB with cerebrovascular-targeted Wnt7a^K190A^ significantly improved neurological outcome of SARS-CoV-2 infection.

## Discussion

SARS-CoV-2, like other respiratory infections, can cause substantial neurological impairment.^3^ However, because such infections cause systemic inflammation, it has been difficult to determine the specific contribution of brain endothelial dysfunction. BBB leakage, leucocyte infiltration, microglial activation, and neuronophagia are well documented in COVID-19 clinical cases and in animal models,^3,11,13,15,16,18,25^ and are plausible causes of neurological signs of disease. Our study provides direct evidence that modulating brain endothelial cell function can mitigate neuroinflammation and neurobehavioural impairment caused by acute SARS-CoV-2 infection.

In this study, our goal was to use an unbiased transcriptomic approach to identify brain endothelial cell gene expression pathways that are dysregulated following respiratory SARS-CoV-2 infection and could be therapeutically targeted. We identified dysregulation of Wnt/β-catenin signalling, which maintains paracellular and transcellular BBB integrity.^19,27-29^ Brain endothelial cell β-catenin signalling is neuroprotective in mouse models for multiple sclerosis by suppressing T cell infiltration, demyelination, and mortality.^29^ Furthermore, cerebrovascular-targeted Wnt ligands protect against glioblastoma and ischemic stroke.^19^ Supported by these findings, we used a gene therapy approach to restore brain endothelial cell Wnt/β-catenin signalling to near physiological levels in a mouse model for acute COVID-19. We determined that cerebrovascular-targeted delivery of Wnt7a^K190A^ prevented BBB permeability, neuroinflammation, and learning and memory deficits in mice infected with SARS-CoV-2.

Viral RNA was detectable in the brain in a subset of infected mice at or near the limit of detection; no infectious virus was recovered from brain. It is possible that this reflects viral RNA fragments deposited in the brain as a result of peripheral infection, or rare abortive infection. We cannot at this time rule out whether neuroinflammation is due to the effect of SARS-CoV-2 in the lung or in the brain itself.

Although our data support a key role for BBB permeability in neuroinflammation and cognitive impairment due to SARS-CoV-2, it is important to note caveats. Because SARS-CoV-2 is a BSL3 pathogen, we were limited in the kinds of neurobehavioural tests we could conduct, because of the practical limitations to conducting behaviour tests within the confines of the biosafety cabinet in the BSL3 suite. We used a pretreatment paradigm, because the gene therapy vector we used to deliver Wnt7a^K190A^ takes ∼3 weeks to achieve maximal transduction of target cells, and our study focused exclusively on acute infection. Future studies are therefore warranted to interrogate the brain endothelial Wnt/β-catenin signalling pathway as a potential therapeutic target to reverse acute or post-acute neurological sequelae of COVID-19, and also to define the specific contributions of distinct components in the brain endothelial Wnt/β-catenin pathway which impact key aspects of cognitive decline in age-related disorders. By design, we focused on evaluating whether intervening with Wnt ligands was beneficial for cerebrovascular and neurological function. Mechanistic studies are also warranted to understand the mechanisms by which respiratory SARS-CoV-2 infection leads to suppressed brain endothelial Wnt/β-catenin signalling and altered neurological function. Due to the central role of Wnt/β-catenin in brain endothelial cell homeostasis, future studies are likely to identify numerous downstream processes by which modulation of the Wnt/β-catenin pathway influences SARS-CoV-2 neuropathogenesis or repair. Furthermore, our transcriptomic analysis yielded numerous dysregulated brain endothelial cell pathways in SARS-CoV-2 infection which although outside the scope of the current investigation are also likely to directly impact BBB function and neurological outcomes of SARS-CoV-2 in parallel with the Wnt/β-catenin signalling pathway.

## Supplementary Material

awae031_Supplementary_Data

## Data Availability

RNA-sequencing data has been deposited on GEO as GSE240903. Additional data are available from the corresponding author.
