# Supplementary material for: Engineered Wnt7a ligands rescue blood–brain barrier and cognitive deficits in a COVID-19 mouse model
Source: Brain. 2024 Feb 2;147(5):1636–43. doi: 10.1093/brain/awae031 (PMC11068107; doi:10.1093/brain/awae031)
Supplement: awae031_Supplementary_Data [file awae031_supplementary_data.pdf]

Supplementary Materials Related To:

Engineered Wnt7a ligands rescue blood brain barrier and cognitive deficits in a COVID-19 mouse model

Troy N. Trevino[1], Avital B. Fogel[1], Guliz Otkiran[1], Seshadri B. Niladhuri[1], Mark A. Sanborn[2], Jacob Class[3], Ali A. Almousawi[1], Benoit Vanhollebeke[4], Leon M. Tai[1], Jalees Rehman[2], Justin M. Richner[3], and Sarah E. Lutz[1]

Materials and methods

SARS-CoV-2 infection

Mouse adapted SARS-CoV-2 MA10 was provided by Ralph Baric[17]. MA10 was propagated and titered on Vero-E6 cells expressing ACE2 and TMPRSS2 (ATCC, CRL1586). Twelve-month-old male C57Bl/6 mice (Jackson Laboratories) on standard light-dark cycles were transferred to Animal BioSafety Level 3 facilities (3-5 mice/cage) >2 days prior to inoculation and randomized to intranasal inoculation with 1×10^4 foci-forming units (FFU) MA10 in 25 µl PBS or vehicle (saline). Behavioral testing, euthanasia, and cell and tissue collection was conducted at 5 days post infection (DPI). Animal studies were approved by the Animal Care Committee (20-107; 20-160).

Modulation of Wnt signaling

AAV-PHP.eB-Wnt7a^{K190A}-P2A-eGFP (AAV:Wnt7a^{K190A}) [19] or AAV:PHP.eB-eGFP (AAV:vector) [19] were administered by retroorbital injection of $2x10^{11}$ viral genomes in 25 µl of PBS 18 days prior to inoculation with SARS-CoV-2 MA10.

Endothelial cell isolation

Mice were transcardially perfused with PBS. Brainstem microvessels were isolated using gradient centrifugation[20], dissociated with collagenase/dispase (Millipore Sigma 10269638001), DNase (Worthington LK003172) for 1 h at 37ºC, and passed through 100 µm cell strainer (PluriSelect USA 43-10100-60). Dissociated microvascular cells were stained for flow cytometric analysis or

were additionally processed with myelin removal beads (Miltenyi 130-069-731) and 3 sequential positive selections with CD31 microbeads (Miltenyi 130-097-418) for isolation of endothelial cell RNA (Qiagen RNeasy Micro Kit 74004). cDNA library preparation used Oligo-dT at 60 million clusters/sample (University of Chicago Genomics Facility).

RNA-sequencing and analysis

Fastq files were quality checked using FastQC (https://www.bioinformatics.babraham.ac.uk/projects/fastqc/) prior to downstream analysis. Reads were aligned using STAR (version 2.7.6a) against the GRCm38 (mm10) genome provided by Ensembl. Count tables were generated using featureCounts (Subread release 2.0.1). Differential expression analysis was performed between testing groups using Deseq2. Clusterprofiler was used for overrepresentation analysis of the differentially expressed genes against the gene ontology database.

Viral RNA quantification

RNA was isolated using RNeasy Mini Kit (Qiagen Cat# 74104) protocol and eluted in a volume of 40uL of RNase free water. Real-time quantitative reverse transcription PCR was performed using TaqMan 1-step RNA to Ct (Thermo Cat# 4392938) with CDC primer/probe kit (IDT - 10006713) against the N1 gene. Samples were analyzed using Viia7 (ThermoFisher) along with Quantstudio 6 and 7 Flex software (ThermoFisher). Genomes/mL were interpolated using Ct values and genomic standard (BEI - NR-52358) run in triplicate.

Infectious virus detection

Forebrain, hindbrain, or olfactory bulbs were homogenized in 1ml or 250µL of PBS, respectively. 100ul of homogenate was added to a monolayer of VeroE6 cells. Images of the monolayer were taken after 4 days of incubation. As a positive control, brain homogenate from a K18-hACE2 mouse at 4 days post infection is included. Images were categorically scored for absence or presence of viral lysis.

Flow cytometry

Cells were sequentially incubated with viability dye (Zombie Green, Biolegend 423111), Fc receptor blockade (Biolegend 101319), and anti-CD31 antibody (BD Biosciences 561073). Cells were fixed (Biolegend 421403) and analyzed (Beckman CytoFLEX S).

Behavioral Assays

Behavior tasks were conducted in a dark biosafety cabinet laminar flow hood in the BSL3 facility. Object familiarization was conducted at 4 DPI between 7:00-10:00PM. Neurobehavioral tests were conducted at 5DPI between 7:00-10:00AM. Novel object recognition was conducted by filming (Logitech C920S HD) mice for ten minutes in white plastic bins 13 inches x 19 inches (Ikea) containing novel and familiar objects. Preference was calculated: (sec investigating novel object)/(sec investigating any object)*100. 50% indicates no preference. For the pole descent assay, the rod of a buret support stand (½ inch diameter, 18 inches length) was mounted on a metal base covered with corn cob bedding. Average latency to descend was measured in two consecutive trials. Falls were scored as failure to descend and excluded from latency averages.

Histology

Mice were perfused with a peristaltic pump. Brains were fixed in 4% paraformaldehyde and paraffin embedded. Antigen retrieval (10 mM Tri-sodium citrate [dihydrate], 0.05% Tween-20, pH 6.0; or Tris-EDTA pH 9.0 for Iba1) was 40 minutes at 98°C. Slides were blocked with 5% goat serum, 0.1% Triton-X 100. Primary antibodies incubated overnight at 4°C at 1:100 included CD3 (Cell Signaling 78588), Glut1 (Abcam ab40084), Fibrinogen (LS Bio LS-C150799-1), Nkd1 (Bioss 19005), CD68 (Biolegend FA-11), and Iba1 (Abcam ab178847). Alexa-fluorophore conjugated secondary antibodies (Invitrogen) were incubated 2h at 22°C. Mounting medium contained DAPI (Ibidi 50011). Microscopy was conducted using Zeiss LSM880 or Leica DMI8 microscopes. Two or three brain sections were analyzed per mouse. From each brain section, microscopy tile scan mosaics were created to capture entire hippocampus or brainstem. CD3+ cells were counted per each individual 20X field, whereas %area measurements were conducted for each mosaic. Quantification was performed using FIJI (NIH).

Statistics

Statistics were conducted using GraphPad Prism 10. Pairwise comparisons used Student's t-test, with Welch's correction when variances were unequal. Pole descent failure was calculated with two-sided Fisher's exact (Chi-square) test. Other tasks were tested with two-way ANOVA; significant interactions were compared by Sidak's multiple comparisons test. Weight loss over time was assessed by two-way repeated measures ANOVA with Tukey's multiple comparisons test. Power analysis indicated that n=5 is required for sensitivity to detect effect size of 0.20 at $p<0.05$ for unpaired two-sided t-test for immunofluorescent staining analysis.

Supplementary Figures:

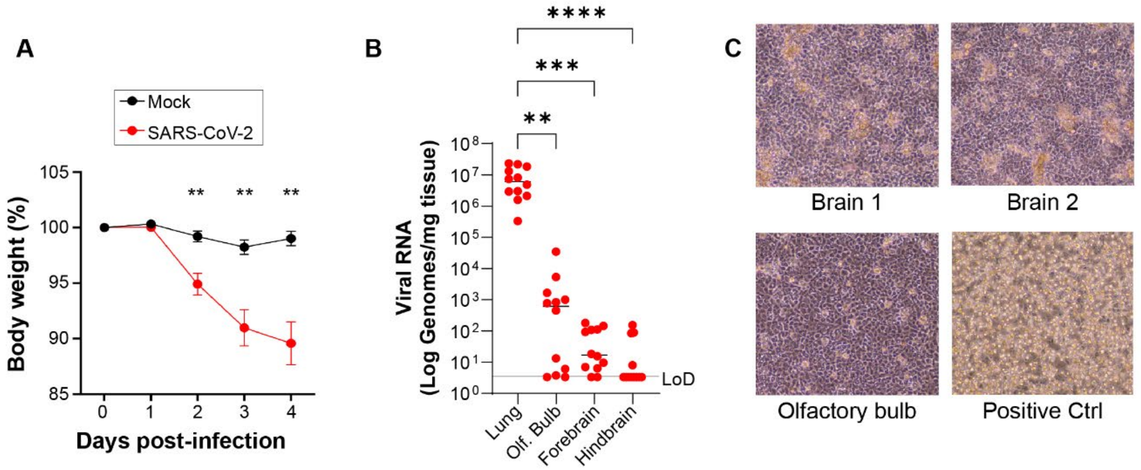

Supplemental Figure 1. Features of MA10 infection. A) Body weight change in mice inoculated with SARS-CoV-2 MA10. Two way repeated measures ANOVA showed an effect of Inoculation [$F_{(1,18)} = 14.09$, $p = 0.0015$], Time [$F_{(1, 25)} = 22.35$, $p < 0.0001$], and Inoculation x Time interaction [$F_{(4, 72)} = 12.26$, $p < 0.0001$]. Post hoc analysis revealed that SARS-CoV-2 infection decreased body weight at 2, 3, and 4 days after infection (** $p < 0.01$). **B)** Viral nucleocapsid RNA detected by QPCR from tissues collected at 5DPI presented as scatterplot with line at median. Dotted line at 3.5 genomes indicates limit of detection (LoD). Significantly more viral RNA was detected in lung than in CNS compartments (olfactory bulb, forebrain [including hippocampus], hindbrain [including brainstem]), assessed by Kruskal-Wallis test with Dunn's multiple comparisons (** p <0.01, *** p <0.001). **C)** No infectious virus detected by cytopathic effect in the brain or olfactory bulb of mice inoculated with SARS-CoV-2 MA10. Brains or olfactory bulbs were homogenized in 1 ml or 250 µL of PBS, respectively. 100 µl of homogenate was added to a monolayer of VeroE6 cells. Images of the VeroE6 monolayer were taken after 4 days of incubation and visually assessed for presence or absence of lysis. As a positive control, brain homogenate from a K18-hACE2 mouse at 4 DPI was included.

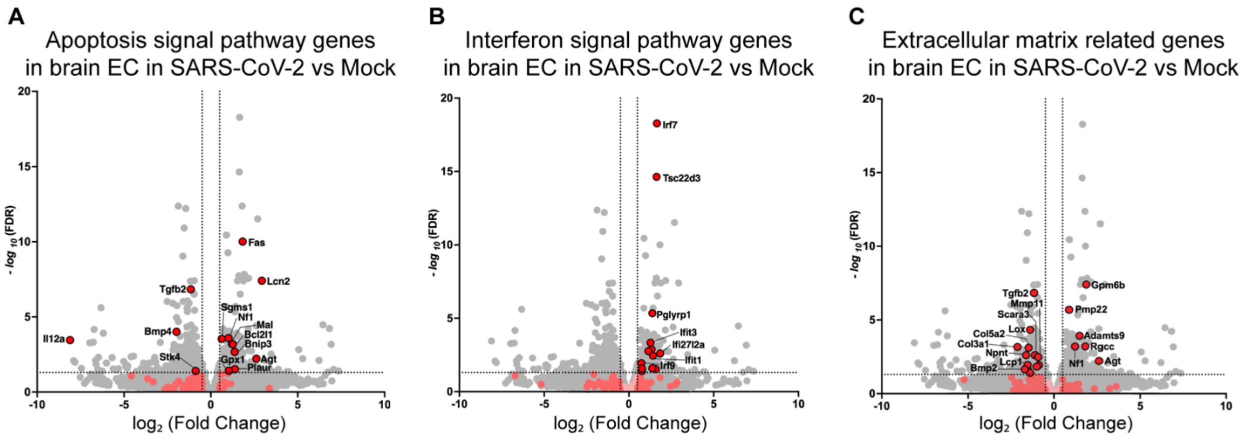

Supplemental Figure 2. Volcano plots depicting differentially expressed genes in selected KEGG pathways in brain endothelial cells of SARS-CoV-2 infected mice as compared to mock infected mice. Differentially expressed genes related to apoptosis (**A**), interferon signal pathway (**B**), and extracellular matrix (**C**) are depicted in red. Notable upregulated transcripts related to apoptosis included Fas and Lcn2 (**A**). Notable upregulated transcripts related to interferon signaling included Irf7 (**B**). Non-differentially expressed pathway genes are depicted in pink. Gray dots represent transcripts that are not part of the indicated pathway.

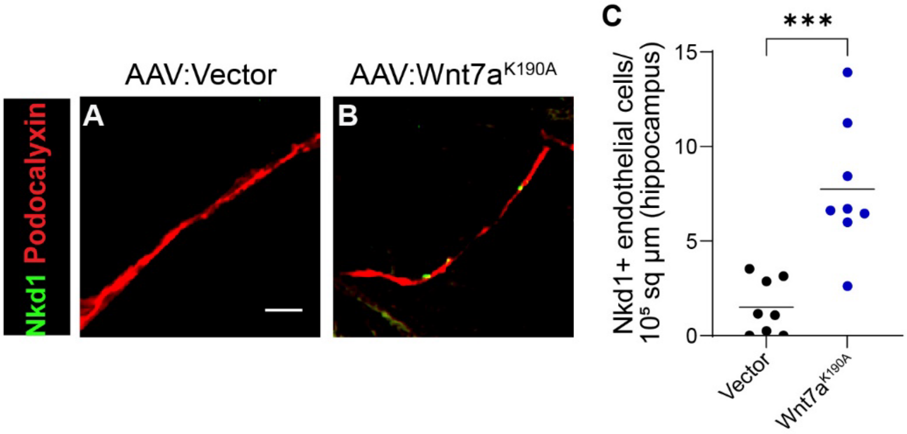

Supplementary Figure 3. Increased expression of the canonical Wnt/β-catenin transcriptional target Nkd1 in brain endothelial cells in SARS-CoV-2 infected mice treated with AAV:Wnt7a^{K190A}. A-B) Immunostaining for Nkd1 (green) and the endothelial marker podocalyxin (red) in hippocampus tissue sections of mice treated with AAV:Vector or with AAV-PHP.eB-Wnt7a^{K190A}-P2A-eGFP. Scalebar indicates 20 µm. **C)** Scatterplot depicting density of Nkd1+ endothelial cells in hippocampus of mice treated with AAV:Vector or with AAV-PHP.eB-Wnt7a^{K190A}-P2A-eGFP. Each dot represents the average value obtained from two to three tissue sections per mouse, $n = 8$ mice/group, with horizontal line to indicate the group mean. Unpaired two-way t-test indicated that AAV-PHP.eB-Wnt7a^{K190A}-P2A-eGFP significantly increased density of Nkd1+ endothelial cells, $***p < 0.001$.

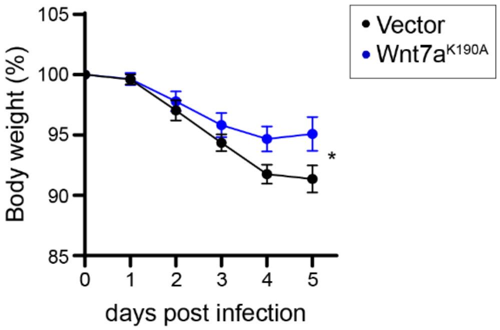

Supplemental Figure 4. Body weight change in mice treated with AAV:Wnt7a^{K190A} or vector control prior to inoculation with SARS-CoV-2 MA10. Two way repeated measures ANOVA showed an effect of Time [$F_{(1.9,27.9)} = 54.71$, $p < 0.0001$], and AAV x Time interaction [$F_{(5,75)} = 3.53$, $p = 0.0064$]. Post hoc analysis revealed significant weight loss in AAV-PHP.eB-Wnt7a^{K190A}-P2A-eGFP treated mice at 4 and 5 days post inoculation with SARS-CoV-2 ($p < 0.05$), and significant weight loss in vector control treated mice at 4 and 5 days post inoculation with SARS-CoV-2 ($p < 0.0005$). Further post hoc analysis revealed that at 5 days post inoculation with SARS-CoV-2, AAV:Wnt7a^{K190A} treated mice had higher body weight than vector control treated mice ($p < 0.05$).

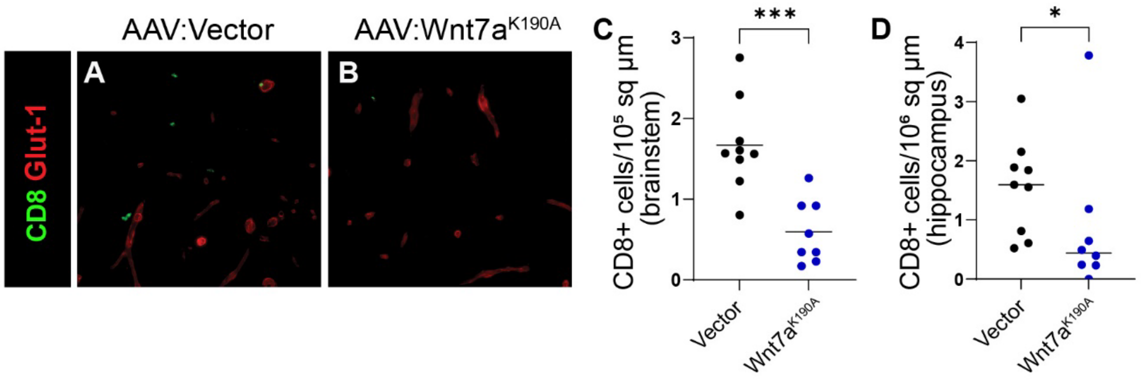

Supplementary Figure 5. Cerebrovascular-targeted engineered Wnt7a^{K190A} decreases CD8+ T cell density in the brains of mice with SARS-CoV-2 infection. Mice were treated with AAV:vector or AAV Wnt7a^{K190A} 18 days prior to inoculation with SARS-CoV-2. Tissue collection was conducted 5 days after SARS-CoV-2 inoculation. (**A-B**) Immunostaining for CD8+ T cells (green) in hippocampus of SARS-CoV-2 infected mice treated with AAV-PHP.eB-Wnt7a^{K190A}-P2A-eGFP or AAV:vector control. Glut1 (red) was used to visualize endothelial cells. **C-D)** SARS-CoV-2 infected AAV-PHP.eB-Wnt7a^{K190A}-P2A-eGFP treated mice had significantly lower density of CD8+ T cells than did AAV:vector control treated mice in brainstem (**C**) and in hippocampus (**D**). Each dot represents the average value obtained from two to three tissue sections per mouse, n = 8 mice/group. Line indicates group mean. *$p < 0.05$, *** $p < 0.001$.

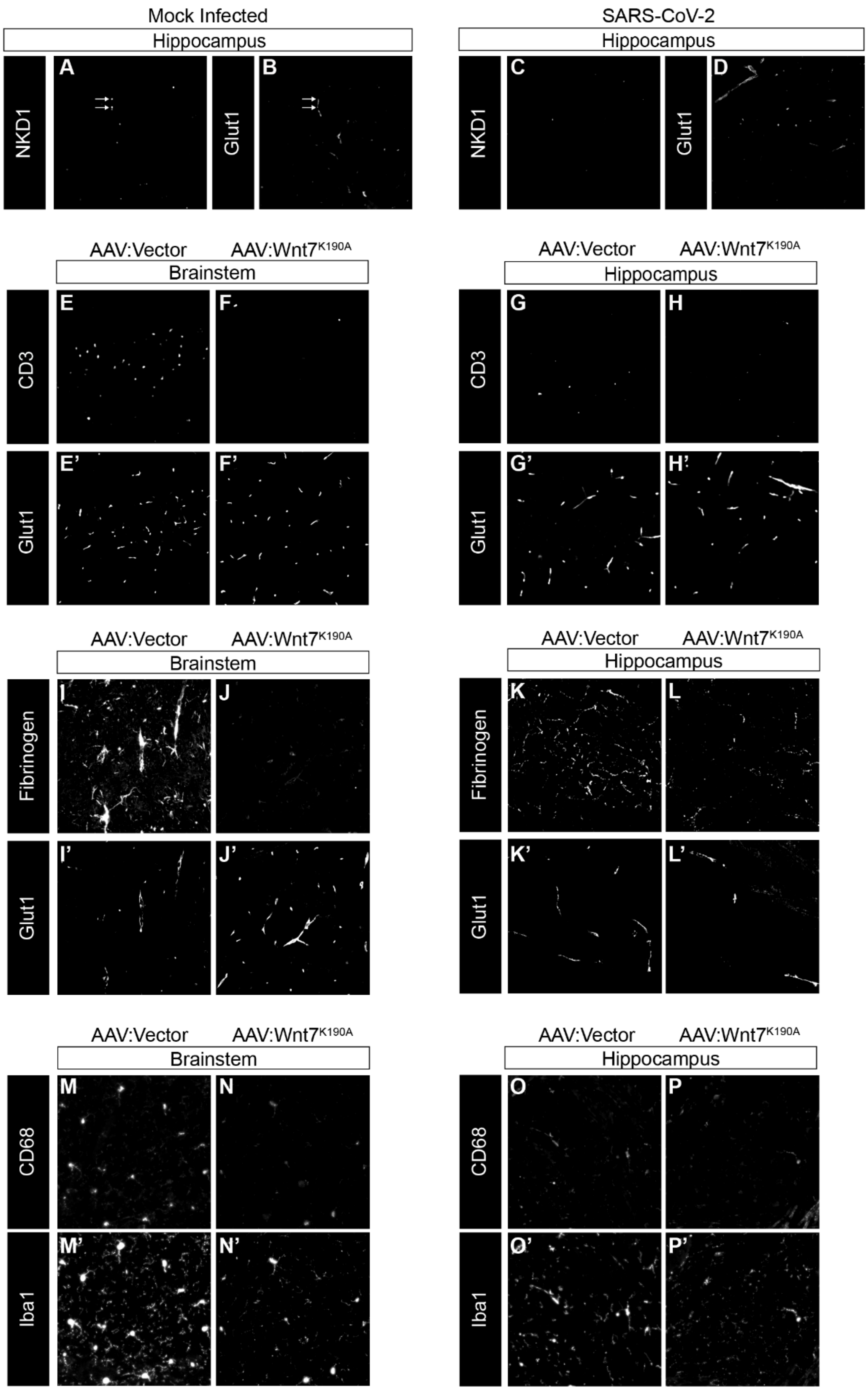

Mock Infected
Hippocampus
SARS-CoV-2
Hippocampus
NKD1
Glut1
A
B
C
D
AAV:Vector
AAV:Wnt7K190A
Brainstem
Hippocampus
CD3
Glut1
E
F
G
H
E'
F'
G'
H'
Fibrinogen
I
J
K
L
I'
J'
K'
L'
CD68
Iba1
M
N
O
P
M'
N'
O'
P'

Supplemental Figure 6. Monochromatic representation of micrographs shown in Figure 2 and Figure 3. A-D) Immunostaining for the β-catenin transcriptional target Nkd1 **(A, C)** in brain sections of SARS-CoV-2 or mock-infected mice. Brain endothelial cells are visualized with Glut-1 **(B, D). E-P)** Mice were treated with AAV-PHP.eB-Wnt7a^{K190A}-P2A-eGFP or AAV-Vector 18 days prior to inoculation with SARS-CoV-2. Immunostaining for CD3+ T cells **(E, F, G, H)** in brainstem **(E, F)** and hippocampus **(G-H)** of SARS-CoV-2 infected mice treated with AAV-PHP.eB-Wnt7a^{K190A} or AAV-PHP.eB vector control. Glut1 **(E'-H')** was used to visualize endothelial cells. SARS-CoV-2 infected AAV-PHP.eB-Wnt7a^{K190A}-P2A-eGFP treated mice had lower density of CD3+ T cells than did AAV-vector treated mice. **I-L)** Immunostaining for fibrinogen **(I - L)** in brainstem **(I, J)** and hippocampus **(K, L)** of SARS-CoV-2 infected mice treated with AAV-PHP.eB-Wnt7a^{K190A}-P2A-eGFP or AAV- vector. Glut1 **(I' - L')** was used to visualize endothelial cells. SARS-CoV-2 infected AAV-PHP.eB-Wnt7a^{K190A}-P2A-eGFP treated mice had lower perivascular fibrinogen than did AAV-vector treated mice in brainstem. **M-P)** Immunostaining for CD68 **(M - P)** and Iba1 **(M' – P')** in brainstem **(M, N)** and hippocampus **(O, P)** of SARS-CoV-2 infected mice treated with AAV-PHP.eB-Wnt7a^{K190A}-P2A-eGFP or AAV-vector. SARS-CoV-2 infected mice had lower CD68+ Iba1+ area.
